# Eicosapentaenoic acid attenuates cigarette smoke-induced lung inflammation by inhibiting ROS-sensitive inflammatory signaling

**DOI:** 10.3389/fphys.2014.00440

**Published:** 2014-11-14

**Authors:** Meng-Han Liu, An-Hsuan Lin, Shing-Hwa Lu, Ruo-Yun Peng, Tzong-Shyuan Lee, Yu Ru Kou

**Affiliations:** ^1^Department of Physiology, School of Medicine, National Yang-Ming UniversityTaipei, Taiwan; ^2^Department of Urology, Taipei City Hospital, Zhong-Xiao BranchTaipei, Taiwan; ^3^Hsin Sheng Junior College of Medical Care and ManagementLongtan Township, Taiwan

**Keywords:** eicosapentaenoic acid, cigarette smoke, lung inflammation, reactive oxygen species, signal transduction, chemokines, lung epithelial cells

## Abstract

Cigarette smoking causes chronic lung inflammation that is mainly regulated by redox-sensitive pathways. Our previous studies have demonstrated that cigarette smoke (CS) activates reactive oxygen species (ROS)-sensitive mitogen-activated protein kinases (MAPKs)/nuclear factor-κB (NF-κB) signaling resulting in induction of lung inflammation. Eicosapentaenoic acid (EPA), a major type of omega-3 polyunsaturated fatty acid, is present in significant amounts in marine-based fish and fish oil. EPA has been shown to possess antioxidant and anti-inflammatory properties *in vitro* and *in vivo*. However, whether EPA has similar beneficial effects against CS-induced lung inflammation remains unclear. Using a murine model, we show that subchronic CS exposure for 4 weeks caused pulmonary inflammatory infiltration (total cell count in bronchoalveolar lavage fluid (BALF), 11.0-fold increase), increased lung vascular permeability (protein level in BALF, 3.1-fold increase), elevated levels of chemokines (11.4–38.2-fold increase) and malondialdehyde (an oxidative stress biomarker; 2.0-fold increase) in the lungs, as well as lung inflammation; all of these CS-induced events were suppressed by daily supplementation with EPA. Using human bronchial epithelial cells, we further show that CS extract (CSE) sequentially activated NADPH oxidase (NADPH oxidase activity, 1.9-fold increase), increased intracellular levels of ROS (3.0-fold increase), activated both MAPKs and NF-κB, and induced interleukin-8 (IL-8; 8.2-fold increase); all these CSE-induced events were inhibited by pretreatment with EPA. Our findings suggest a novel role for EPA in alleviating the oxidative stress and lung inflammation induced by subchronic CS exposure *in vivo* and in suppressing the CSE-induced IL-8 *in vitro* via its antioxidant function and by inhibiting MAPKs/NF-κB signaling.

## Introduction

Cigarette smoking is the major etiologic factor in the development of chronic obstructive pulmonary disease (COPD), which is characterized by chronic lung inflammation (Chung and Adcock, 2008). Cigarette smoke (CS)-induced lung inflammation is regulated by a complex mechanism that involves various types of cells and a number of inflammatory mediators (Barnes, 2004; Chung and Adcock, 2008). For example, because the lung epithelium is a target for direct insult by CS, chemokines such as interleukin-8 (IL-8), which are released from lung epithelial cells, play an important role in the initiation and progression of lung inflammation (Mio et al., 1997; Mossman et al., 2006; Thorley and Tetley, 2007; Moretto et al., 2008; Tang et al., 2011; Liu et al., 2014; Wu et al., 2014). The induction of inflammatory mediators by CS in various types of lung cells is mainly regulated by redox-sensitive signaling pathways (Mossman et al., 2006; Rahman and Adcock, 2006). Initially, CS may increase the intracellular levels of reactive oxygen species (ROS) in lung cells, mainly via activation of NADPH oxidase (Mossman et al., 2006; Rahman and Adcock, 2006; Cheng et al., 2009; Lin et al., 2010; Tang et al., 2011; Liu et al., 2014; Wu et al., 2014). Subsequently, this increased in intracellular ROS may serve as the trigger to activate various ROS-sensitive signaling pathways, such as the mitogen-activated protein kinases (MAPKs) and a number of downstream transcriptional factors, such as nuclear factor-κB (NF-κB); this ultimately increase the production of inflammatory mediators (Mossman et al., 2006; Rahman and Adcock, 2006; Tang et al., 2011; Liu et al., 2014; Wu et al., 2014). Due to the involvement of the ROS-sensitive signaling pathways, dietary antioxidant therapies targeting oxidative stress should be beneficial and help to improve CS-induced lung inflammation (Biswas et al., 2013).

Omega-3 polyunsaturated fatty acids (n-3 PUFAs) are present in significant amounts in marine-based fish and fish oil and have long been known to possess a wide range of beneficial effects, including the treatment of various forms of chronic inflammatory diseases (Chapkin et al., 2009; Giudetti and Cagnazzo, 2012; Davidson, 2013). Recently, there has been a growing interest in the beneficial effect of eicosapentaenoic acid (EPA), a major type of n-3 PUFAs (Davidson, 2013). For example, a clinical trial has revealed that highly purified EPA prevents the onset of cardiovascular events in hypercholesterolaemic patients (Yokoyama et al., 2007). Additionally, EPA has been shown to suppress inflammatory responses to stimuli other than CS in a number of animal models (Okabe et al., 2011; Jia et al., 2012; Poudyal et al., 2013; Schuster et al., 2014) and in various cell types (Moon et al., 2007; Mickleborough et al., 2009; Mullen et al., 2010; Wang et al., 2010; Jinno et al., 2011; Jung et al., 2012; Magee et al., 2012; van den Elsen et al., 2013). Furthermore, EPA has been reported to possess antioxidant activity when prescribed to treat patients (Mahmoudabadi and Rahbar, 2014) or when used as *in vitro* (Richard et al., 2008; Kusunoki et al., 2013; van den Elsen et al., 2013) or *in vivo* preparations (Okabe et al., 2011; Palaniswamy et al., 2014). Thus, the antioxidant and anti-inflammatory properties of EPA make it a potential drug for the treatment of CS-induced lung inflammation. However, this possibility remains to be proven.

The aims of this study were, firstly, to investigate the antioxidant and anti-inflammatory effects of EPA on CS-induced lung inflammation and, secondly, to determine any therapeutic mechanisms underlying the beneficial effects of EPA. We used an established murine model of subchronic CS exposure (Tang et al., 2011; Wu et al., 2014) to assess the inhibitory effects of EPA on oxidative stress and various indices of lung inflammation. Additionally, we used primary human bronchial epithelial cells (HBECs) to determine the suppressive effects of EPA on the CS extract (CSE)-mediated increases in intracellular ROS, activation of the ROS-sensitive inflammatory signaling pathways, and the induction of IL-8.

## Methods

### Reagents

Antibodies (Abs) and ELISA kits to measure IL-8, macrophage inflammatory protein 2 (MIP-2), monocyte chemoattractant protein-1 (MCP-1) and keratinocyte chemoattractant (KC) were purchased from R&D Systems (Minneapolis, MN, USA). Malondialdehyde (MDA) was purchased from Abcam (Cambridge, MA, USA). Antibodies against ERK, JNK, phospho-ERK, phospho-JNK, p65, and Histone H1 were obtained from Santa Cruz Biotechnology (Santa Cruz, CA, USA). Mouse antibody against α-tubulin, EPA (purity ≥99%) and the 3-(4,5-dimethylthiazol-2-yl)-2,5 diphenyltetrazolium bromide (MTT) assay kit were purchased from Sigma-Aldrich (St. Louis, MO, USA). The EnzyChrom NADP^+^/NADPH assay kit was obtained from BioAssay Systems (Hayward, CA, USA). The membrane-permeable probes hydroethidine (HE) and dichlorofluorescein diacetate (DCFH-DA) were purchased from Molecular Probes (Eugene, OR, USA).

### Murine model of subchronic CS exposure and EPA treatment

All animal experiments were approved by the Animal Care and Use Committee of the National Yang-Ming University. The murine model of subchronic CS exposure has been described in detail previously (Tang et al., 2011; Wu et al., 2014). Briefly, male C57BL/6J mice at the age of 8 weeks (National Laboratory Animal Center, Taipei, Taiwan) were randomly divided into four groups (7 mice/group) for exposure to air or CS. These mice received daily treatment with EPA (50 mg/kg) or saline (vehicle control) by gastric gavage during the 4-week exposure. The mice formed four groups, namely Air, Air+EPA, CS, and CS+EPA. Animals were given *ad libitum* access to food and water, and their average body weights did not vary among the study groups at the end of the 4-week exposure. For each CS exposure, the mice were placed in an exposure chamber (40 × 30 × 20 cm; Shin Chen EEC-1, Taipei, Taiwan) and 750 ml of fresh CS generated from 1.5 cigarettes (Marlboro Red Label; 10.8 mg nicotine and 10.0 mg tar per cigarette) was delivered to the chamber. The CS passed out of the chamber via four exhaust holes (1 cm) on the side panels. During the exposure, the mice were conscious and breathed spontaneously in the chamber for 10 min. After exposure, the mice were transferred to a new cage and allowed to inspire air normally. The mice were exposed at 10:00 and 16:00 each day for 4 weeks. The control animals underwent identical procedures in another chamber but were only exposed to air. For each CS exposure, the particle concentration inside the exposure chamber was about 625 mg/m^3^ initially, but decreased overtime due to the fact that the CS passed out of the chamber via the exhaust holes (Wu et al., 2014). The HbCO levels immediately after the 10 min exposure protocol for air-exposure and CS-exposure mice were 0.4 and 32%, respectively (Wu et al., 2014).

### Preparation of bronchoalveolar (BALF) and lung tissues

At the end of each experiment, the mice were euthanized with CO_2_ and a middle thoracotomy was performed. The left lung was ligated and the right lung was lavaged four times with 0.4 ml of warm PBS containing a complete protease inhibitor cocktail (Roche Diagnostics, Mannheim, Germany). The BALF samples were then centrifuged at 350 × g for 5 min at 4°C, and the supernatant of the first lavage fluid was stored at −80°C for later analysis of total protein using Bio-Rad protein assay reagent (Bio-Rad Laboratories, Hercules, CA, USA). The cell pellets of the BALF samples were re-suspended in PBS for cell counting. Furthermore, the right lung was then stored at −80°C for subsequent analysis. The left lung was fixed with 4% paraformaldehyde and embedded in paraffin.

### Histological assessment

Formalin-fixed, paraffin-embedded tissue blocks were cut into 8-μm sections. Sections were deparaffinized, rehydrated, and then underwent haematoxylin and eosin (H&E) staining and were viewed under a microscope (Motic TYPE 102M, Xiamen, China). The histological assessments were conducted by a pathologist who was blinded to the treatment. Each histological characteristic was scored on a scale of 0 (normal) to 5 (maximal). The lung inflammatory scores were categorized according to the sum of the score for infiltration cell numbers and for damage levels, including thickening of alveolar walls and epithelium, as well as increases in peribronchial and perivascular cuff area.

### Determining lung levels of chemokines and oxidative stress

The concentrations of MIP-2, MCP-1, and KC in the lung tissue samples were measured using ELISA kits according to the manufacturer's instructions. In addition, levels of MDA, a product of lipid peroxidation, in the lung tissue samples were measured by assay kits according to the manufacturer's instructions and the results served as a biomarker for oxidative stress (Ardite et al., 2006).

### Preparation of CSE

CSE was freshly prepared on the day of the experiment as previously described (Tang et al., 2011; Wu et al., 2014). In brief, 1000 ml of the smoke generated from two burning cigarettes (Marlboro Red Label) without filters were sucked at a constant flow rate (8 ml/s) into a syringe and then bubbled into a tube containing 10 ml serum-free medium. The CSE solution was sterilized using a 0.22-μm filter (Millipore, Bedford, MA, USA) and the pH was adjusted to 7.4. The optical density of the CSE solution was determined by measuring the absorbance at 302 nm (Yamaguchi et al., 2007) or 320 nm (Facchinetti et al., 2007), which, in reality, were found to show little difference between different preparations. This CSE solution was considered 100% CSE and was further diluted with serum-free medium to the desired concentrations, which were then used to treat HBECs for different times.

### Cell culture

HBECs (Cascade Biologics, Portland, OR, USA) were cultured in epithelial cell growth medium (F12K medium; Cascade Biologics, USA) containing 10% fetal bovine serum (FBS), 100 U/ml penicillin, 100 μg/ml streptomycin, and 0.25 μg/ml amphotericin B (Biological Industries, Kibbutz Beit Haemek, Israel) at 37°C in an incubator with 5% CO_2_.

### Cell viability assay

Cell viability was measured by the MTT assay as described previously (Liu et al., 2005). Briefly, cells were incubated with or without EPA for 24 h and 100 μl of MTT (0.5 mg/ml in medium) were then added. The cells incubated with control medium were considered 100% viable.

### Measurement of intracellular ROS levels

The membrane-permeable probes HE and DCFH-DA were used to assess levels of ROS using methods that have been described previously (Liu et al., 2005). Oxidation of HE by ROS, preferentially superoxide, forms red fluorescent ethidium (ETH) (Benov et al., 1998), whereas oxidation of DCFH-DA by ROS, particularly hydrogen peroxide, yields fluorescent 2,7-dichlorofluorescein (DCF) (Myhre et al., 2003). For the purpose of these experiments, HBECs were incubated in culture medium containing 10 μM HE or 20 μM DCFH-DA at 37°C for 30 min. Then, cell medium was replaced with fresh medium. After stimulation with CSE for 30 min, cells were washed and detached with trypsin/EDTA, and the fluorescence intensity of the cells was analyzed by use of a multilabel counter (PerkinElmer, Waltham, MA) at 518 nm excitation and 605 nm emission for ETH, and at 488 nm excitation and 530 nm emission for DCF. Images of the cells were also obtained by examining them using a Nikon TE2000-U florescence microscope (Tokyo, Japan).

### Determination of NADPH oxidase activity

The activity of NADPH oxidase was measured using an EnzyChrom™ NADP^+^/NADPH assay kit according to the manufacturer's instructions. This assay kit measures the change in NADP^+^/NADPH ratio in cellular lysate samples and reflects the relative NADPH oxidase activity of the samples tested.

### Western BLOT analysis

Aliquots of cell lysates or tissue lysates were separated by 8–12% SDS-PAGE and then transblotted onto Immobilon™-P membrane (Millipore). After being blocked with 5% skim milk, the blots were incubated with various primary antibodies, and then appropriate secondary antibodies. The specific protein bands were detected using an enhanced chemiluminescence kit (PerkinElmer), which was followed by the quantification using ImageQuant 5.2 software (Healthcare Bio-Sciences, Philadelphia, PA, USA).

### Statistical analysis

The results are presented as mean ± s.e.m. Statistical evaluations involved One-Way ANOVA followed by Dunnett's test or Fisher's least significant difference procedure for multiple comparisons as appropriate. Differences were considered statistically significant at *p* < 0.05.

## Results

### Effect of EPA on inflammatory manifestations in mice

**Figure 1:**
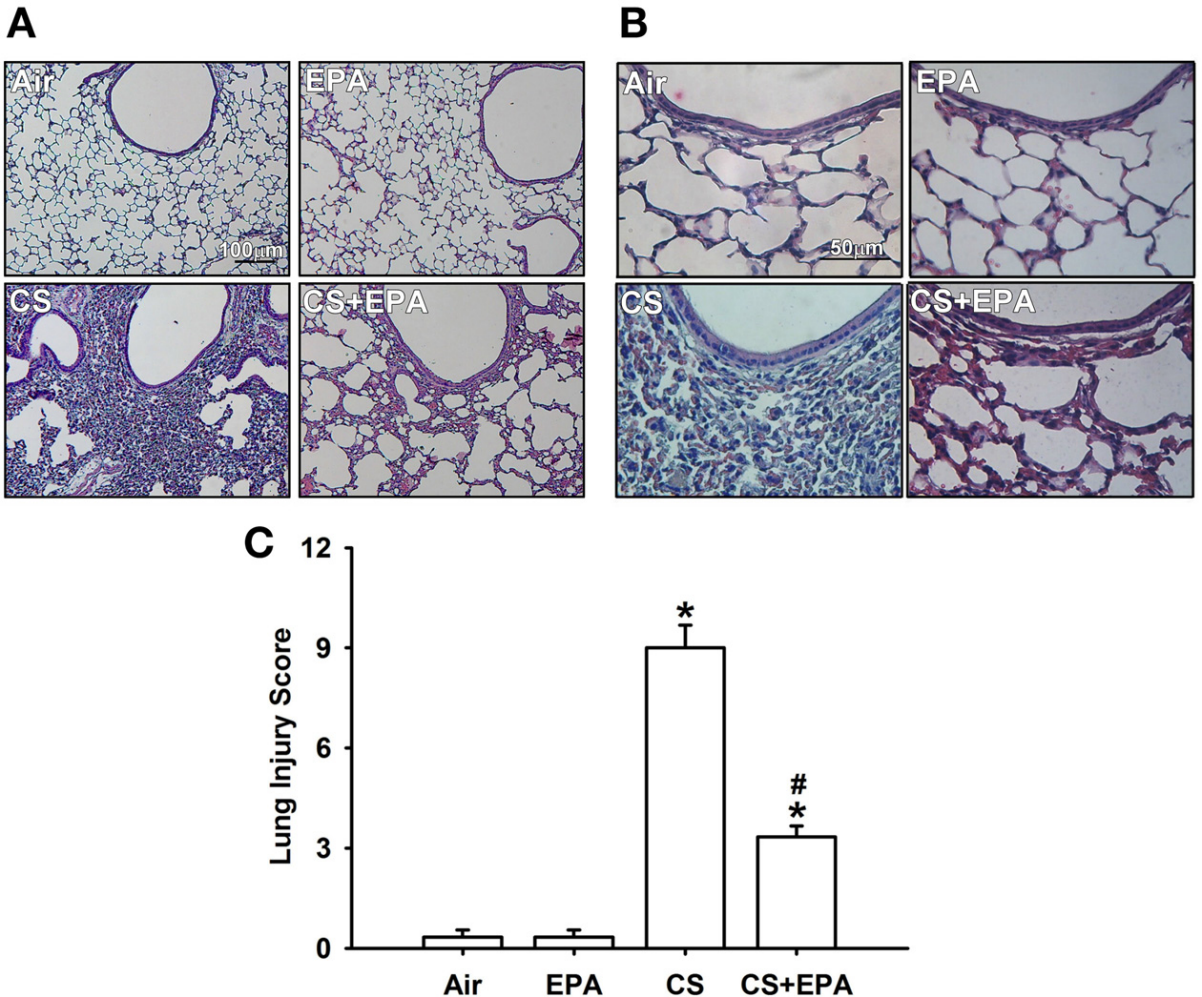
**Eicosapentaenoic acid (EPA) attenuates cigarette smoke (CS)-induced lung inflammation in mice**. Four groups of mice were subchronically exposed to air or CS for 4 weeks. Two of the four study groups received daily treatment with EPA (50 mg/kg body weight) or vehicle (50% alcohol and 50% DMSO) by gastric gavage during the 4-week exposure. **(A,B)** Representative images of H&E stained lung sections. The magnifications of each **(A,B)** are 100X and 400X, respectively. **(C)** Lung inflammatory scores were calculated according to the sum of the levels of cell infiltration and damage levels as assessed from the lung sections. Data in each group are mean ± s.e.m. from 6 mice. ^*^p < 0.05 vs. the air-exposure group; ^#^*p* < 0.05 vs. the CS-exposure group with vehicle treatment.

**Figure 2:**
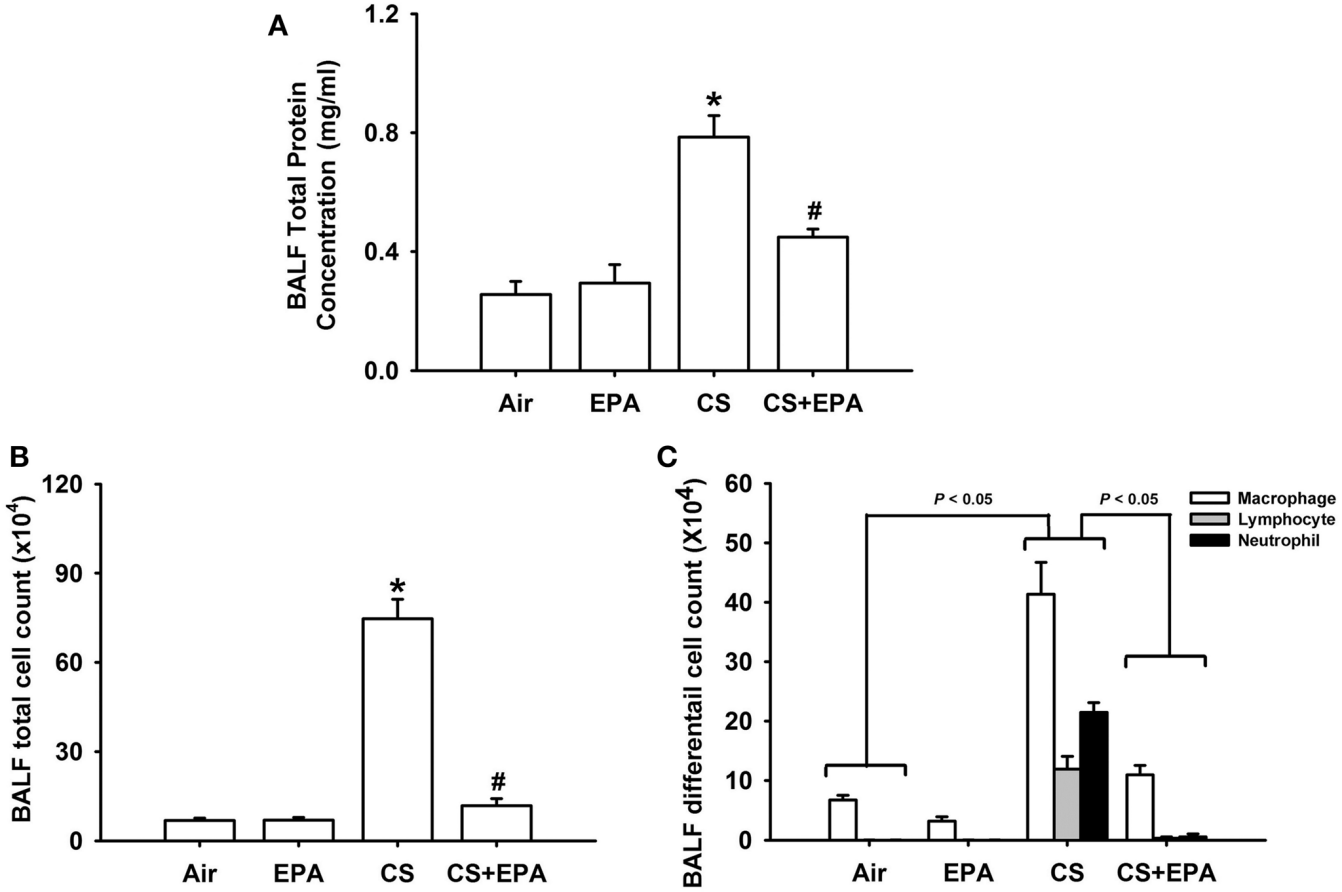
**Eicosapentaenoic acid (EPA) attenuates cigarette smoke (CS)-induced increases in total protein content **(A)**, total cell count **(B)** and differential cell count **(C)** in bronchoalveolar lavage fluid (BALF)**. These indices were measured and served as indications of lung inflammation. Data in each group are mean ± s.e.m. from 6 mice. ^*^p < 0.05 vs. the air-exposure group; ^#^*p* < 0.05 vs. the CS-exposure group with vehicle treatment. See the legend of Figure 1 for detailed information on each study group.

Exposure of mice to CS for 4 weeks resulted in the development of lung inflammation as evidenced by the histological evaluation of the H&E stained lung sections. The inflammatory manifestations included extensive infiltration of inflammatory cells, thickening of the alveolar walls and the presence of abnormal re-epithelialization in the CS-exposure mice (Figures 1A,B); these changes were found to be less in the CS-exposure mice that underwent EPA treatment (Figures 1A,B). Comparisons of the group data in terms of lung inflammatory scores confirmed this difference in the degree of histopathological manifestations between the CS-exposure mice with and without EPA treatment (Figure 1C). Additionally, compared to the air-exposure mice, the CS-exposure mice were found to show increases in total protein levels (Figure 2A), total cell counts (Figure 2B) and differential cell counts (Figure 2C) in BALF. All of these inflammatory indices were significantly alleviated in the CS-exposure mice that underwent EPA treatment (Figure 2). These inflammatory manifestations were not found in the air-exposure mice that underwent EPA treatment (Figures 1, 2).

### Effect of EPA on increases in chemokines and oxidative stress in mice

**Figure 3:**
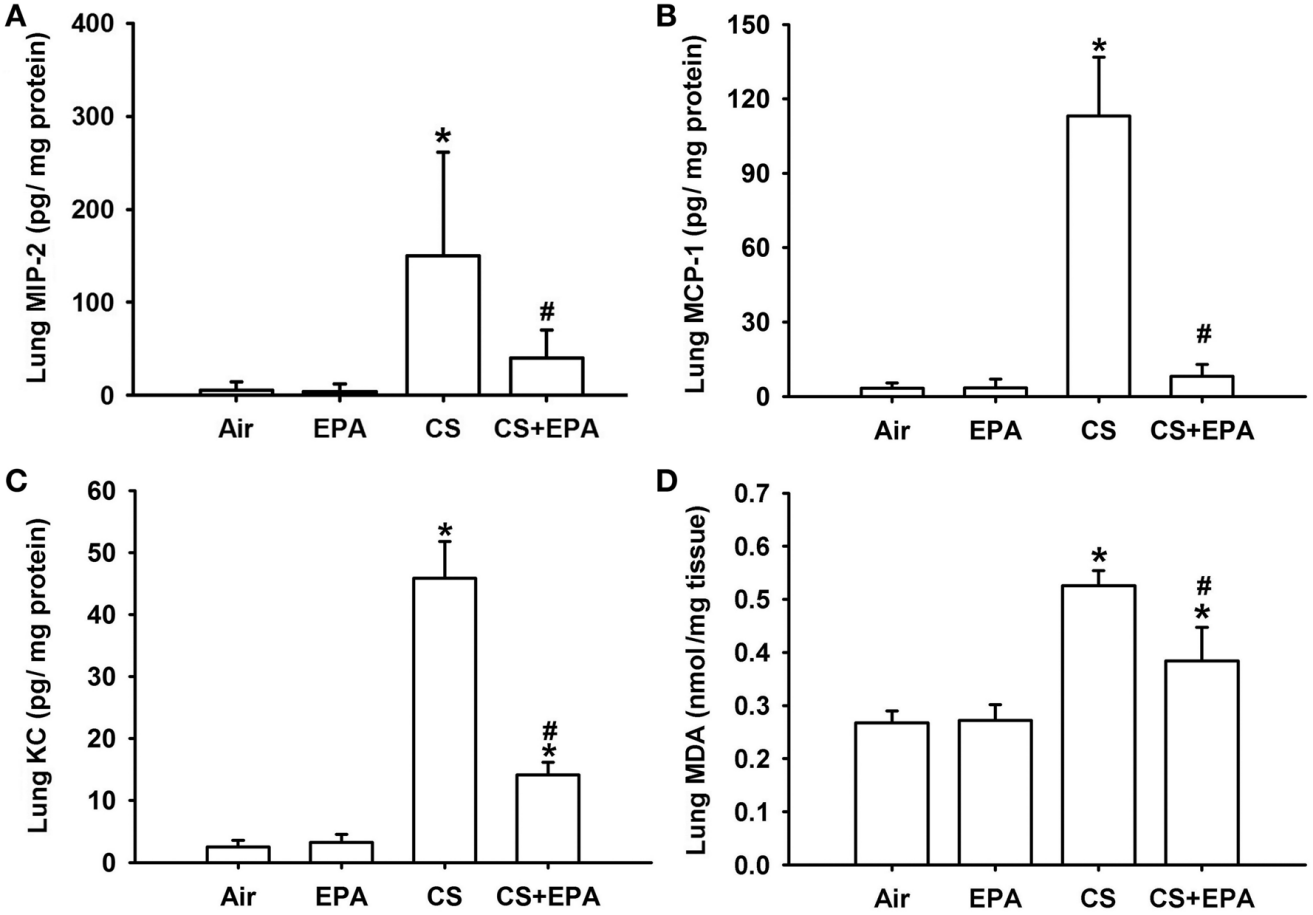
**Eicosapentaenoic acid (EPA) attenuates cigarette smoke (CS)-induced increases in pro-inflammatory chemokines and malondialdehyde in lung tissues sampled from mice**. Levels of macrophage inflammatory protein 2 (MIP-2) **(A)**, monocyte chemoattractant protein-1 (MCP-1) **(B)** and keratinocyte chemoattractant (KC) **(C)** in lung tissues were analyzed by ELISA. Levels of malondialdehyde (MDA) **(D)** were measured by an assay kit and served as an indication of oxidative stress. Data in each group are mean ± s.e.m. from 6 mice. ^*^p < 0.05 vs. the air-exposure group; ^#^*p* < 0.05 vs. the CS-exposure group with vehicle treatment. See the legend of Figure 1 for detailed information on each study group.

Compared to the air-exposure mice, exposure of mice to CS for 4 weeks resulted in increases in the levels of MIP-2 (Figure 3A), MCP-1 (Figure 3B), and KC (Figure 3C) in the lung tissue samples. The increases in the levels of these chemokines were found to be significantly reduced in the CS-exposure mice that had underwent EPA treatment (Figure 3). Additionally, compared to the air-exposure mice, exposure of mice to CS led to an increase in MDA levels, a biomarker of oxidative stress, in the lung tissue samples, which was found to be significantly attenuated in the CS-exposure mice that underwent EPA treatment (Figure 3D). EPA treatment did not produce notable changes in levels of these chemokines and of MDA in the air-exposure mice (Figure 3).

### Effect of EPA on the induction of IL-8 in HBECs

**Figure 4:**
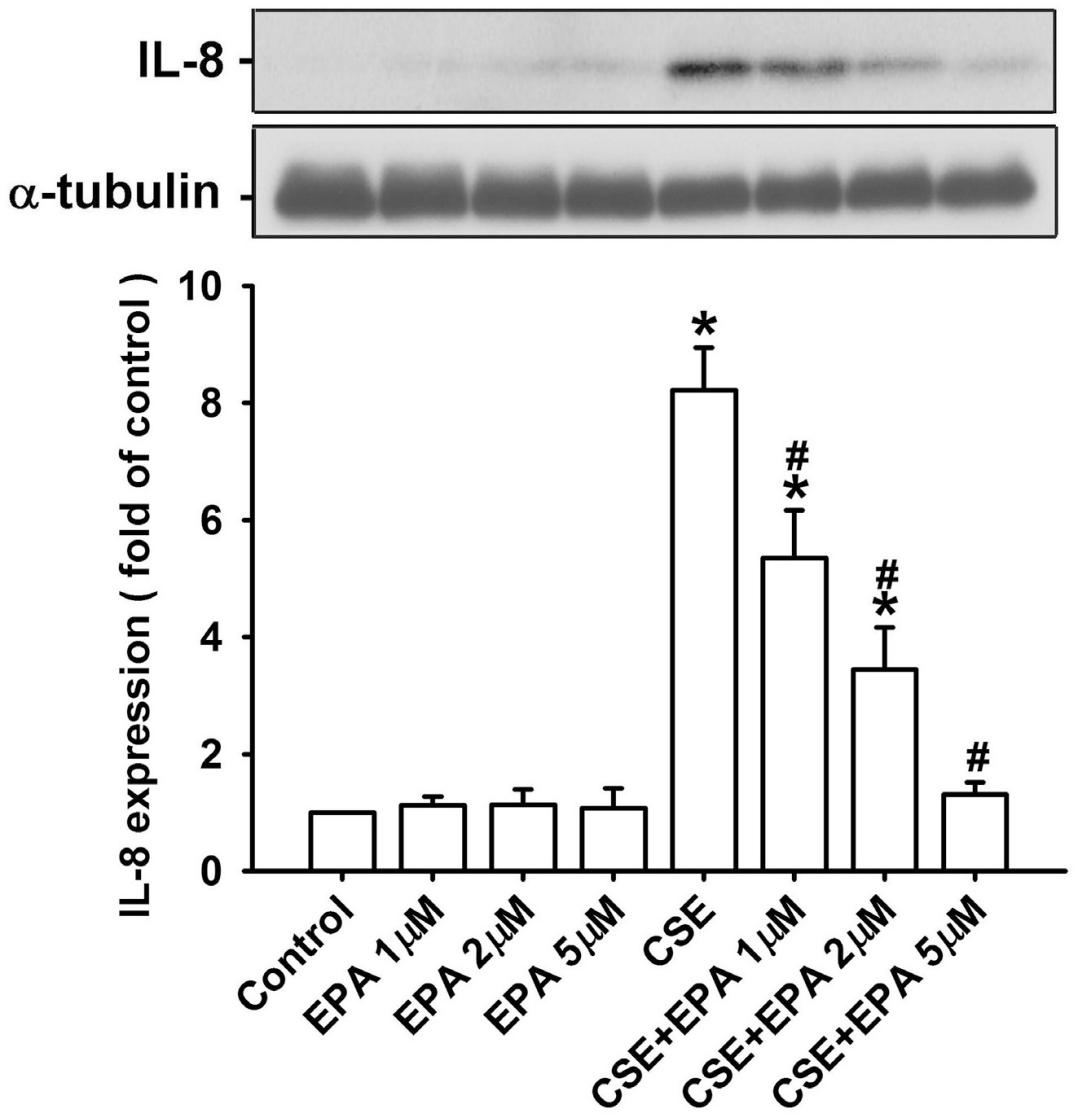
**Eicosapentaenoic acid (EPA) dose-dependently attenuates the induction of IL-8 by cigarette smoke extract (CSE) in human bronchial epithelial cells**. Cells were incubated with medium alone or 3% CSE for 24 h with pretreatment with vehicle or various concentrations (1, 2, and 5 μM) of EPA. Protein levels of IL-8 in the cell lysates were analyzed by Western blotting. Data in each group are mean ± s.e.m. from five independent experiments. ^*^p < 0.05 vs. control (vehicle without CSE stimulation). ^#^*p* < 0.05 vs. CSE without EPA pretreatment.

In addition, we used HBECs as an *in vitro* model to study the therapeutic mechanism of EPA. Based upon the concentration and time-response relationships reported previously (Tang et al., 2011; Liu et al., 2014; Wu et al., 2014), exposure of HBECs to 3% CSE for 24 h was employed as the standard challenge to induce IL-8. Pretreatment with various concentrations of EPA (0, 1, 2, and 5 μM) concentration-dependently attenuated the induction of IL-8 by CSE, whereas pretreatment with EPA in cells without CSE stimulation failed to alter the expression of IL-8 (Figure 4). The results obtained from MTT assay indicated that exposure of HBECs to EPA at a concentration of 5 μM for 24 h did not alter cell viability (102.4 ± 3.5% of control). Based on the above, we employed 5 μM EPA as the standard treatment.

### Effect of EPA on the increase in intracellular ROS in HBECs

**Figure 5:**
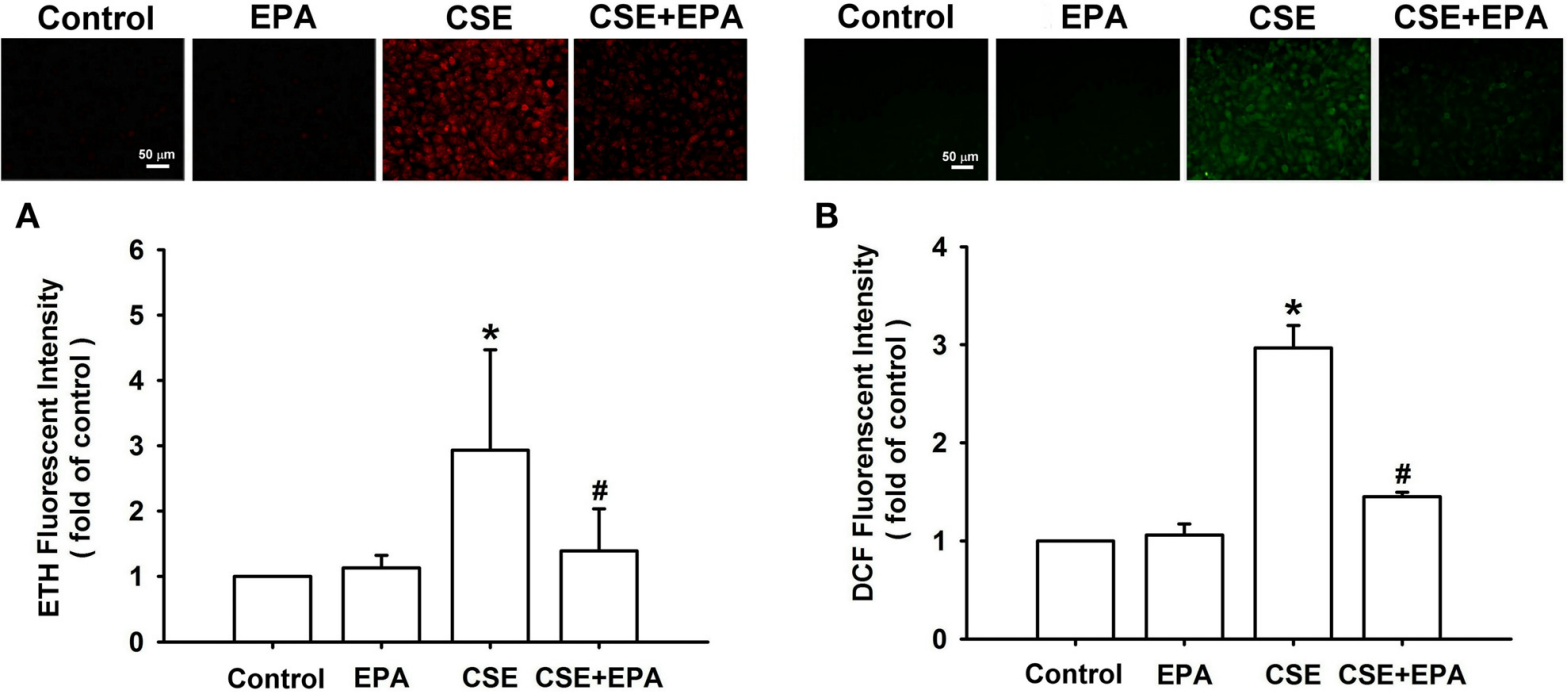
**Eicosapentaenoic acid (EPA) attenuates increases in intracellular levels of reactive oxygen species (ROS) induced by cigarette smoke extract (CSE) in human bronchial epithelial cells**. Cells were exposed to medium alone or 3% CSE for 30 min with pretreatment with EPA (5 μM) or its vehicle. After exposure, the cells were collected for the measurement of intracellular ROS levels. Levels of ROS were measured by HE/ETH **(A)** and DCFH-DA/DCFH **(B)** fluorescent probes. Data in each group are mean ± s.e.m. from five independent experiments. ^*^p < 0.05 vs. control (vehicle without CSE stimulation). ^#^*p* < 0.05 vs. CSE without EPA pretreatment.

**Figure 6:**
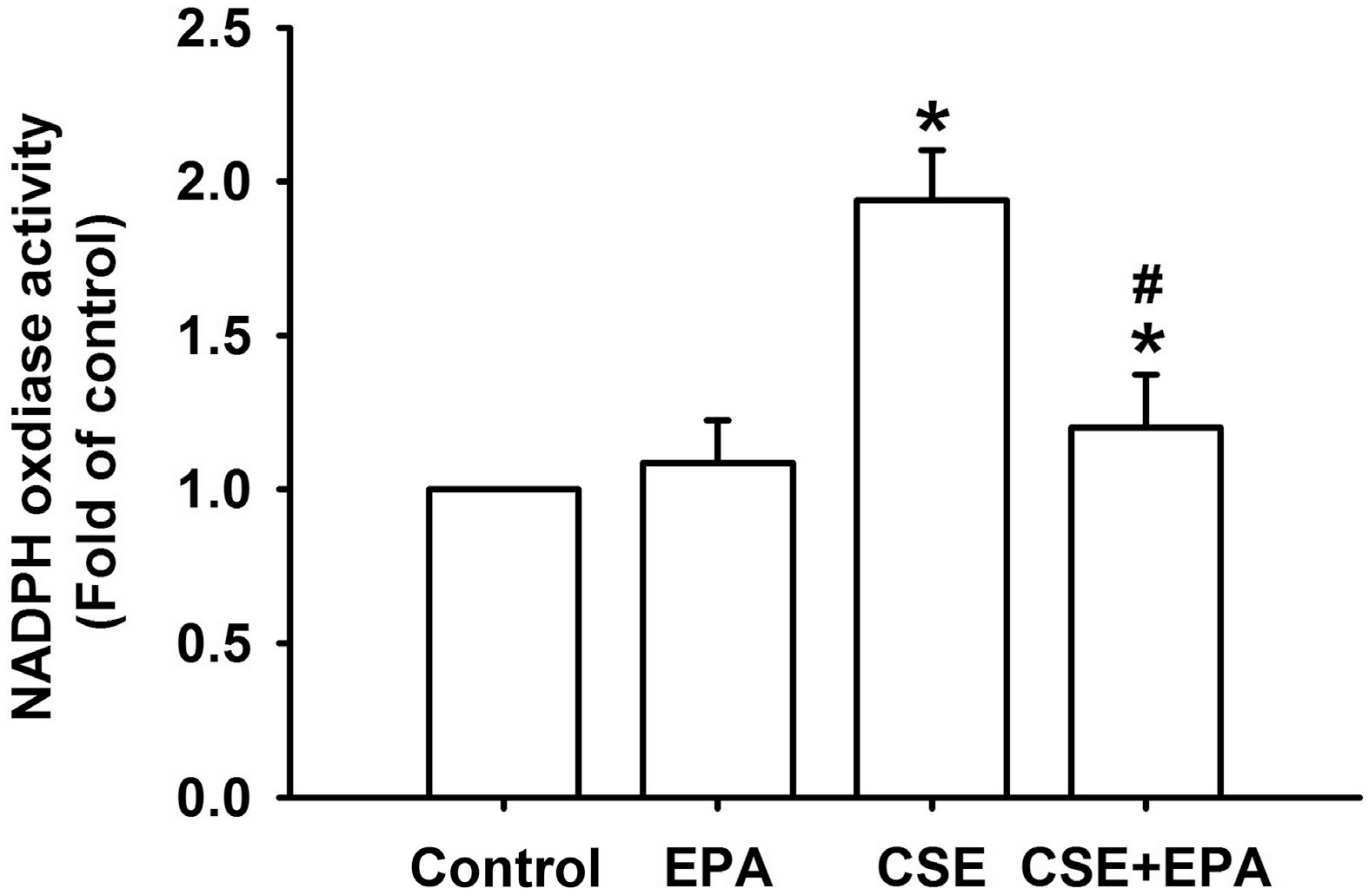
**Eicosapentaenoic acid (EPA) attenuates the increase in NADPH oxidase activity induced by cigarette smoke extract (CSE) in human bronchial epithelial cells**. Cells were exposed to medium alone or to 3% CSE for 15 min with EPA (5 μM) or its vehicle. NADPH oxidase activity was measured by NADP^+^/NADPH assay. Data in each group are mean ± s.e.m. from five independent experiments. ^*^p < 0.05 vs. control (vehicle without CSE stimulation). ^#^*p* < 0.05 vs. CSE without EPA pretreatment.

Increases in intracellular ROS, via activation of NADHP oxidase, are an important trigger for the induction of IL-8 by CSE in HBECs (Tang et al., 2011; Wu et al., 2014). Compared to control cells, exposure of HBECs to 3% CSE for 30 min resulted in increases in intracellular levels of both superoxide (Figure 5A) and hydrogen peroxide (Figure 5B). The CSE-induced increases in intracellular ROS were prevented by pretreatment with EPA (Figure 5). Further analysis revealed that exposure of HBECs to 3% CSE for 15 min led to an increase in the activity of NADPH oxidase and this was found to be suppressed by pretreatment with EPA (Figure 6). Pretreatment with EPA in cells without CSE stimulation failed to alter the levels of intracellular ROS (Figure 5) and the activity of NADPH oxidase (Figure 6).

### Effect of EPA on the activation of MAPKS/NF-κB signaling

**Figure 7:**
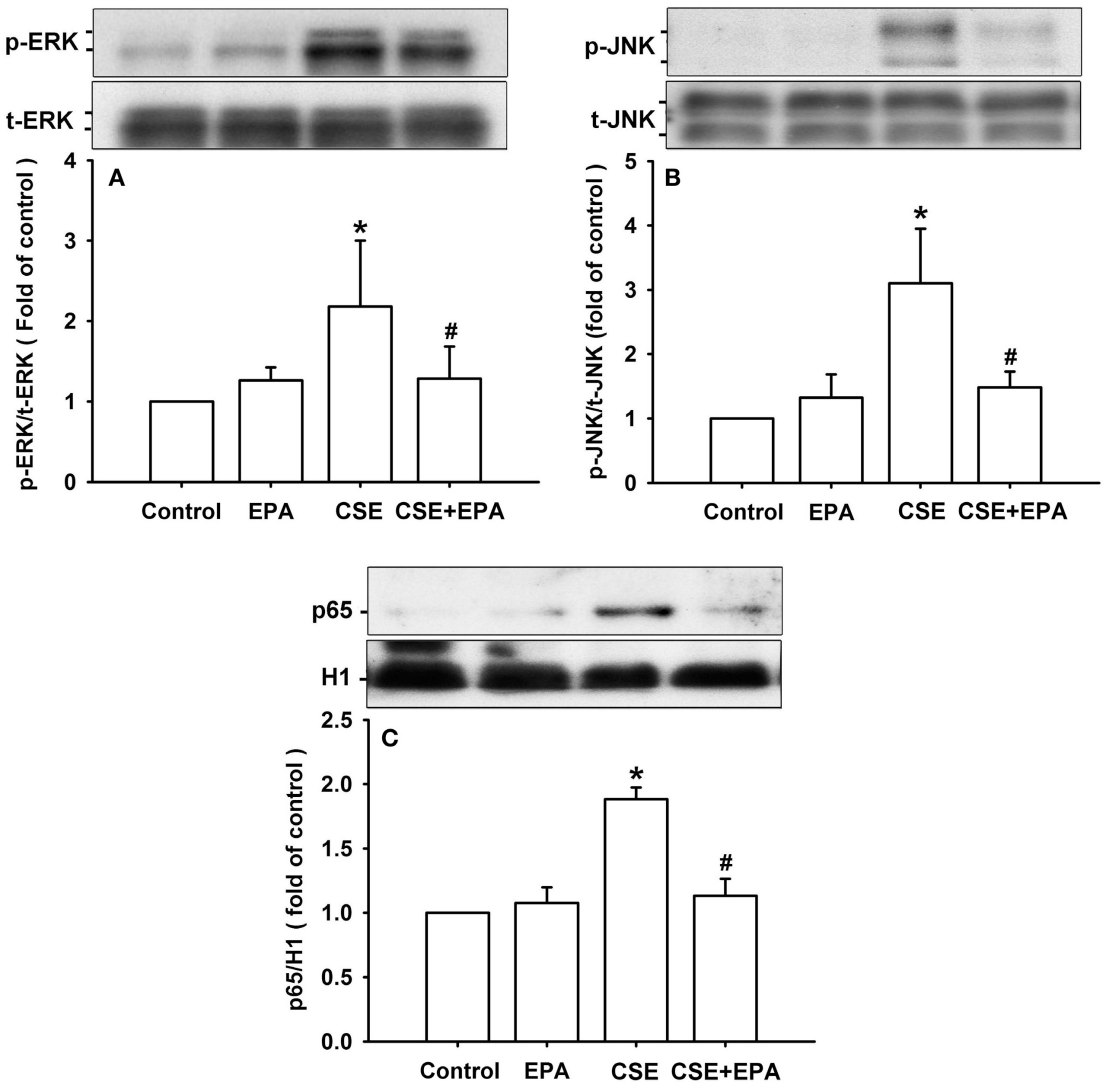
**Eicosapentaenoic acid (EPA) attenuates the activation of ERK, JNK and NF-κB signaling by cigarette smoke extract (CSE) in human bronchial epithelial cells**. Cells were exposed to medium alone or 3% CSE for 6 **(A,B)** or 12 h **(C)** with pretreatment with EPA (5 μM) or its vehicle. Protein expression was analyzed by Western blotting. ^*^p < 0.05 vs. control (vehicle without CSE stimulation). ^#^*p* < 0.05 vs. CSE without EPA pretreatment.

Activation of ERK, JNK, and NF-κB is known to be a ROS-sensitive signaling pathway that is vital to the induction of IL-8 by CSE in HBECs (Tang et al., 2011; Liu et al., 2014; Wu et al., 2014). Compared to control cells, exposure of HBECs to 3% CSE for 6 h resulted in increases in the amount of both phosphorylated ERK (Figure 7A) and phosphorylated JNK (Figure 7B). Furthermore, exposure of HBECs to 3% CSE for 12 h resulted in an increase in the amount of NF-κB p65 subunit present in the nuclei of cells (Figure 7C). Such CSE-induced activation of the MAPKs/NF-κB signaling was significantly attenuated by pretreatment with EPA (Figure 7). Pretreatment with EPA in cells without CSE stimulation failed to alter the expression of these proteins (Figure 7).

## Discussion

Our *in vivo* study demonstrates clearly that subchronic CS exposure of mice for 4 weeks causes lung inflammation as evidenced in lung tissue samples by histopathological manifestations as well as several inflammatory indices, such as an increase in BALF total cell count, an increase in BALF differential cell count an increase in total protein and increased levels of various inflammatory chemokines, including MIP-2, MCP-1, and KC. All these are potent chemokines related to the initiation and progression of lung inflammation induced by CS (Yoshida and Tuder, 2007) and, among these chemokines, MIP-2 and KC are murine homologs of IL-8. Subchronic CS exposure also was found to cause an increase in oxidative stress as reflected by the increased level of MDA in the lungs. All of these CS-induced events are similar to those described previously (Barnes, 2004; Thorley and Tetley, 2007; Tang et al., 2011; Liu et al., 2014; Wu et al., 2014). Importantly, all of these CS-induced pathophysiological events were found to be suppressed by daily treatment with EPA, suggesting that EPA has both anti-inflammatory and antioxidant functions against CS-induced lung inflammation *in vivo*. To investigate the therapeutic mechanism of EPA, we then employed HBECs to study the induction of IL-8 by CSE; this is because IL-8 produced by lung epithelial cells is important to the induction of lung inflammation by CS (Mossman et al., 2006; Thorley and Tetley, 2007; Moretto et al., 2008; Tang et al., 2011; Liu et al., 2014; Wu et al., 2014). Our *in vitro* study demonstrates that exposure of HBECs to CSE sequentially activated NADPH oxidase, increased intracellular ROS level, activated both MAPKs and NF-κB, and induced IL-8. Previous studies have revealed that the CSE-induced increase in intracellular ROS is mediated through activation of NADHP oxidase and this is an important trigger for the ROS-sensitive MAPKs/NF-κB signaling pathway that leads to the induction of IL-8 in HBECs (Tang et al., 2011; Liu et al., 2014; Wu et al., 2014). Notably, all of these CSE-induced consequences were suppressed by pretreatment with EPA, indicating that the beneficial effect of EPA may be mediated through its antioxidant activity and via the inhibition of the ROS-sensitive inflammatory signaling.

Our study appears to be the first to report that EPA has both antioxidant and anti-inflammatory properties against CS-induced lung inflammation. These two beneficial activities of EPA have been suggested by previous studies that have focused on stimuli other than CS insult. Indeed, supplementation of EPA has been shown to suppress the chronic inflammation in several animal models including ovalbumin-induced asthma in mice (Schuster et al., 2014), high-fat diet-induced metabolic syndrome in rats (Poudyal et al., 2013), high fat-cholesterol diet-induced fibrotic steatohepatitis in rats (Jia et al., 2012) and ischemic brain injury in gerbils (Okabe et al., 2011). Using an *in vitro* preparation, administration of EPA has been demonstrated to attenuate inflammatory responses to stimuli in macrophages (Mickleborough et al., 2009; Mullen et al., 2010; Wang et al., 2010; Jinno et al., 2011; Jung et al., 2012), mast cells (van den Elsen et al., 2013), myoblasts (Magee et al., 2012), and microglia (Moon et al., 2007). In addition to its anti-inflammatory activity, administration of EPA has been reported to suppress ROS generation *in vivo* (Richard et al., 2008; van den Elsen et al., 2013). EPA also possesses antioxidant activity in type 2 diabetic patients (Mahmoudabadi and Rahbar, 2014), in rats exposed to organic pollutants (Palaniswamy et al., 2014) and against oxidative stress in adipocytes (Kusunoki et al., 2013). Thus, our findings are in good agreement with the above previously reported observations.

There are at least three possibilities to explain the suppressive effect of EPA on the CS-induced lung inflammation in our model system. First, EPA may prevent the CS-induced increase in intracellular ROS via suppression of the activation of NADPH oxidase (Morre et al., 2010) as indicated in this study or via scavenging ROS (Richard et al., 2008). EPA has also been reported to up-regulate antioxidant enzymes (Kusunoki et al., 2013; Mahmoudabadi and Rahbar, 2014; Palaniswamy et al., 2014). While the latter mechanism may be possible explanation of our *in vivo* study, it is unlikely to be true for our *in vitro* study. This is because the effective treatment time with EPA (30 min) is too short to suppress the increase in intracellular ROS perhaps via the up-regulation of an antioxidant system. Since the induction of lung inflammatory mediators by CS is mainly regulated by ROS-sensitive signaling pathways (Mossman et al., 2006; Rahman and Adcock, 2006; Tang et al., 2011; Wu et al., 2014), it is plausible that the anti-inflammatory effect of EPA is linked to its antioxidant function. Secondly, we should also include the possibility that EPA directly interferes with the activation of this signaling pathway because this has been suggested by other investigators (Mullen et al., 2010; Jia et al., 2012; Magee et al., 2012; van den Elsen et al., 2013). Thirdly, EPA may give rise to lipid mediators such as E-series resolvins, which are known to have potent anti-inflammatory and pro-resolution activity (Seki et al., 2009). In this context, docosahexaenoic acid (DHA), which is another n-3 PUFA, has been well studied regarding its antioxidant and anti-inflammatory properties (Chapkin et al., 2009; Giudetti and Cagnazzo, 2012; Davidson, 2013). While many studies have shown qualitative and quantitative differences in responses to EPA and DHA (Russell and Bürgin-Maunder, 2012), certain metabolic derivatives of DHA have been shown to have beneficial effects against CS-induced lung inflammation that are similar to those reported in this study (Hsiao et al., 2013; Cipollina et al., 2014). Of note, Shahar et al. reported that the prevalence odds of COPD in smokers were inversely related to the plasma level of DHA, but not EPA (Shahar et al., 1999). However, these investigators (Shahar et al., 1999) interpreted this discrepancy with caution because human body can metabolize EPA to DHA and some of the EPA in the diet may eventually be metabolized to DHA.

Our *in vivo* studies demonstrate that the EPA-mediated attenuation of CS-induced lung inflammation is associated with a reduction of lung oxidative stress, which is consistent with our *in vitro* findings. It is still unknown whether the beneficial effects of EPA are limited to the induction of chemokines in lung epithelial cells or also apply to other cell types such as leukocytes. If the latter is true, the target cells for EPA will not be confined to only the lung epithelial cells because the EPA was given systemically to the mice in this study. Since we used HBECs for *in vitro* studies and mice for *in vivo* studies, the therapeutic mechanisms of EPA should be extrapolated carefully to *in vivo* model. Additionally, although EPA was administered orally by gavage, no measurements of EPA blood levels and its variability were made. We also did not attempt to study the dose-dependence in the *in vivo* effects of EPA. Of note, our *in vitro* study shows that the dose of EPA used did not produce any notable cytotoxicity as revealed by the assay for cell viability. In our *in vivo* study, the dose of EPA (50 mg/kg) is within the range of mean daily intakes from food in humans (EFSA Panel on Dietetic Products, Nutrition and Allergies, 2012) and was chosen to avoid possible adverse effects. Clinical trials have reported that a daily consumption of EPA up to 500 mg (Mahmoudabadi and Rahbar, 2014) or 1800 mg (Yokoyama et al., 2007) is safe when treating patients with chronic inflammatory diseases.

In summary, our findings suggest a novel role for EPA regarding the alleviation of oxidative stress and lung inflammation induced by subchronic CS exposure *in vivo*, and the suppression of the CSE-induced IL-8 *in vitro* by inhibiting MAPKs/NF-κB signaling, possibly via its antioxidant function. Our findings support the possibility of using EPA to ameliorate lung inflammation in smokers.

## Author contributions

Meng-Han Liu, An-Hsuan Lin, Shing-Hwa Lu, and Ruo-Yun Peng conducted the studies, analyzed the data and interpreted experimental results. Meng-Han Liu, An-Hsuan Lin wrote the paper. Tzong-Shyuan Lee and Yu Ru Kou led the project, interpreted the data and wrote the paper.

### Conflict of interest statement

The authors declare that the research was conducted in the absence of any commercial or financial relationships that could be construed as a potential conflict of interest.
